# Onlay platelet-rich fibrin membrane versus free gingival graft in increasing the width of keratinized mucosa around dental implants: A split-mouth randomized clinical study

**DOI:** 10.34172/japid.2022.013

**Published:** 2022-08-24

**Authors:** Zeinab Al-Diasty, Samah El-Meadawy, Ahmed S Salem, Bassant Mowafey

**Affiliations:** ^1^Department of Periodontology, Faculty of Dentistry, Mansoura University, Egypt; ^2^Department of Oral Medicine and Periodontology, Faculty of Dentistry, Mansoura University, Egypt; ^3^Department of Oral and Maxillofacial Surgery, Faculty of Dentistry, Mansoura University, Egypt; ^4^Department of Oral Diagnosis and Oral Radiology, Faculty of Dentistry, Mansoura University, Egypt

**Keywords:** Free gingival graft (FGG), gingival augmentation, platelet-rich fibrin

## Abstract

**Background.** This study aimed to compare the use of the platelet-rich fibrin membrane (PRF) versus the free gingival graft (FGG) during the second stage of the dental implant to increase the amount of keratinized mucosa around dental implants.

**Methods.** Fifteen patients with bilaterally missing teeth and deficient width of the keratinized mucosa (KM) were recruited for a spit-mouth randomized controlled trial. After implant placement on the control sides, onlay FGG was used, whereas, on the other side (study side), onlay PRF membranes were applied to augment the KM. One month and three months after augmentation, the increase in keratinized mucosa width, bone level around the implants, and soft tissue health were evaluated and compared. The shrinkage percentage was also calculated for both grafts.

**Results.** There was a significant increase in the width of KM in the FGG and PRF groups; however, it was observed that FFG resulted in significantly better results than PRF, with no significant difference in peri-implant soft tissue health or bone level.

**Conclusion.** Within the limitations of this study, it was concluded that the onlay PRF membrane could increase the keratinized mucosa width around dental implants with the advantages of a lower surgical time and less postoperative discomfort and pain for the patients in comparison to the FGG. However, FGG had a significantly higher ability to augment and increase keratinized mucosa around dental implants.

## Introduction

 Dental implants are associated with multiple challenges, such as achieving and maintaining stable soft tissue and bone around osseointegrated dental implants. It is well established in much research that at least a 2-mm width of keratinized mucosa (KM) around a dental implant is mandatory to maintain gingival health.^1,2^ Even in patients with good oral hygiene and on regular implant maintenance therapy, implants with a reduced width of <2 mm of keratinized mucosa were more prone to lingual plaque accumulation and bleeding as well as buccal soft tissue recession over five years.^3^

 Free gingival graft (FGG) taken from the palate as a donor site is the gold standard to augment the deficient keratinized mucosa.^4^ FGG is used to guard against hard and soft tissue problems developed after implant rehabilitation.^5^ However, the most obvious problems for patients with FGG are the donor site morbidity, pain, a change in diet, paresthesia, herpetic lesions, mucocele, arteriovenous shunts, and excessive bleeding.^6^FGG has limitations, both regarding the quantitative (volume augmentation) and qualitative outcomes (aesthetic integration, surface, color, and scarring).^7^

 Many procedures and materials have been developed to augment keratinized mucosa around dental implants to overcome these problems. PRF is a 2nd generation platelet concentrate introduced by Choukroun et al^8^ in 2001. It can be obtained by a simple and inexpensive procedure that does not need biochemical blood handling. Its 3-dimensional fibrin network promotes neovascularization, accelerates wound closure, and fast cicatricial tissue remodeling.^9^ PRF provides autologous growth factors that stimulate cell migration and proliferation. Because PRF is produced without using any additive, the fibrin polymerization occurs physiologically, resulting in a similar fibrin network as the one formed during natural healing ^9,10^

 This study was based on the null hypothesis that assumes that there is no difference between the onlay PRF membrane study sides and the onlay free gingival graft control sides. Therefore, the present study aimed to compare the use of onlay PRF membrane versus onlay free gingival graft during the second stage of dental implant placement to increase the amount of keratinized mucosa around the dental implant.

## Methods

###  Study population

**Figure 1:**
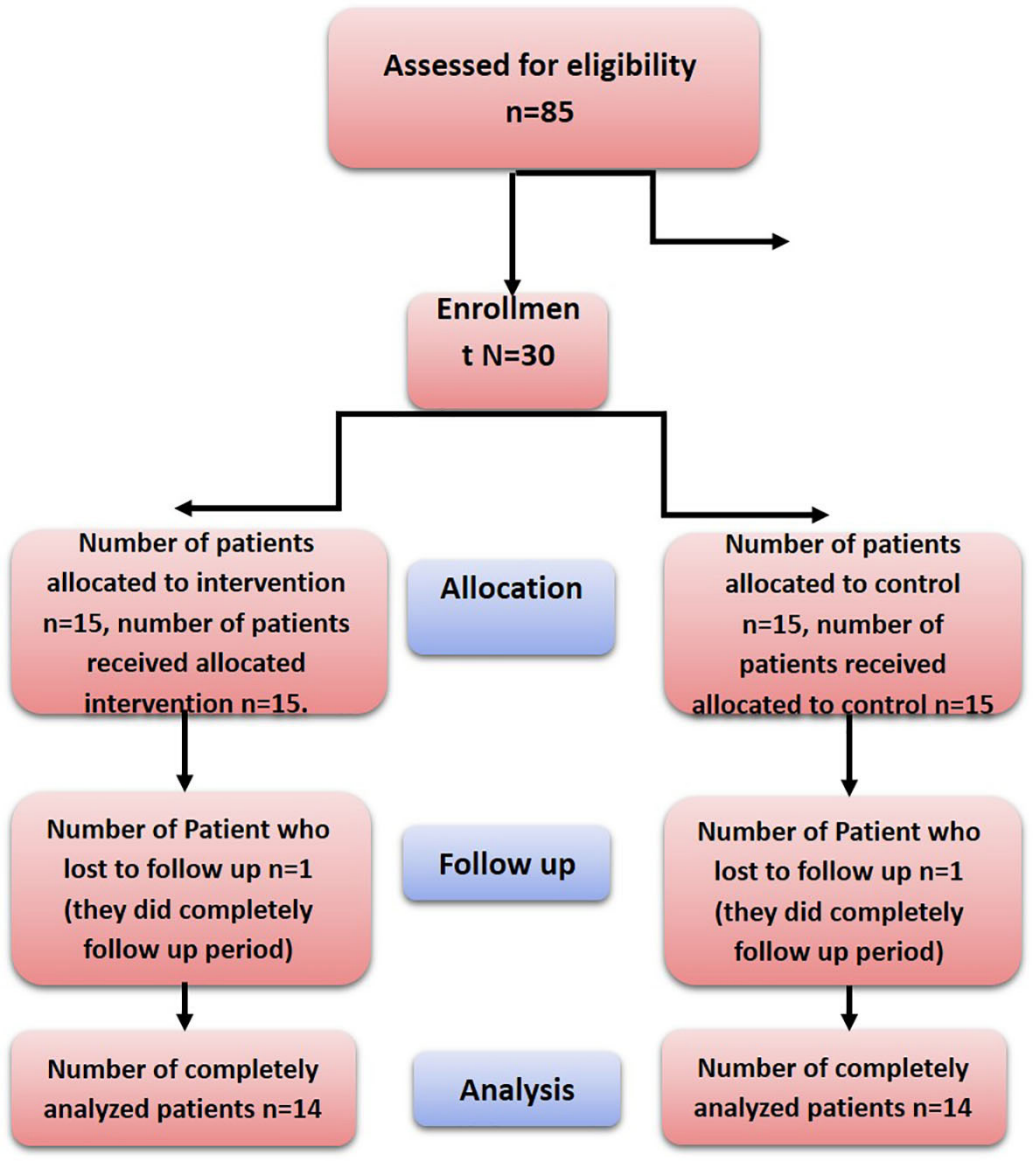
Flowchart of the study.

 The present study was conducted following the seventh revision of the Helsinki Declaration in 2013 and was approved by the Institutional Review Board (IRB) of the Faculty of Dentistry, Mansoura University, Egypt. The patients were selected during their visits to the clinic of the Periodontology Department at the Faculty of Dentistry, Mansoura University, Egypt, from July 2018 to June 2021. Fifteen patients (8 females and 7 males) were included in the study, and their age range was 25–35 years. The number of implants placed was 30 (Figure 1).

 The patients had at least one missing tooth bilaterally that needed replacement with dental implants. Enrolled patients signed informed consents before they participated in this clinical trial. The included patients had narrow keratinized mucosa measuring ≤2 mm, healthy systemic condition, and adequate inter-arch space. Some patients had had their teeth extracted, and the cause of extraction was not related to either advanced periodontal diseases or systemic osteoporotic diseases. There was a mesiodistal distance of at least 7 mm between the adjacent teeth, a bone width of at least 6 mm buccolingually at the edentulous site, and a bone height of at least 10 mm. The teeth adjacent to the study site had no signs of periodontal bone loss or significant soft tissue loss.

 Exclusion criteriaincluded psychiatric problems, unrealistic expectations, uncooperative patients, and a history of abnormal parafunctional habits, e.g., bruxism and clenching, patients with a smoking habit, poor oral hygiene, and the presence of any soft tissue or bony pathological lesions at the intended implant site.

###  Study design

 The sample size of the study was determined based on the null hypothesis of the study, which stated that the test group (PRF membrane) and the control group (FGG) were not equal in their outcomes. The desired study power was 95%, with a confidence level of 95%. The G power software (version 3.1.9) was used, and the required sample size was determined at 13 patients (26 edentulous sites). The selected sample size was increased to 15 patients to account for the possible attrition of candidates without the possibility of outcome changes in the study.

 This study was a split-mouth randomized clinical trial. The patients’ randomization was carried out by one of the senior residents in the department, not involved in the study and not aware of any related treatment protocol. The patients were randomly distributed into 15 patients in the test group (onlay PRF) or 15 patients in the control group (onlay FGG) in a split-mouth design via a randomization table by a computer-generated randomization list (SPSS v23.0). The cases were operated by the same operator who was not involved in the evaluation. The assessor did all the evaluation steps and was completely blinded to the treatment protocol.

###  Surgical procedure

 Preoperative cone-beam computed tomography (CBCT) was used to evaluate the residual bone at the intended implant insertion site. Accordingly, the ideal implant size was selected. The patient was instructed to rinse with 0.12% chlorhexidine (CHX) mouthwash (Hexitol, Arab Drug Company, Egypt) as antimicrobial prophylaxis three times daily starting two days before surgery. The patient was given 1 g of antibiotic (875 mg amoxicillin/125 mg clavulanic acid) (Megamox, Julphar, Egypt) one hour before surgery.

 Infiltration anesthesia was carried out using 4% Articaine HCL with 1:100.000 adrenaline (Artinibsa 4%, Inibsa, Spain). Implant fixtures were placed into their planned surgical sites according to the standard drilling protocol of stage 1 surgery for a dental implant. The patients were instructed to continue the antibiotic course for seven days after surgery. Analgesic sodium diclofenac 50 mg (Cataflam, Novartis, Egypt) was described tid for two days, then as required. The patients were also instructed to rinse with a mouthwash containing 0.12% chlorohexidine gluconate solution three times a day for two weeks. The patients were recalled, and the sutures were removed 7‒10 days after implant placement.

 In the second stage of surgery, the patients were recalled after three months to expose the implant and to apply soft tissue augmentation procedures. All the patients were instructed to rinse with 0.12% CHX mouthwash for 30 seconds. After local anesthesia was infiltrated, a partial thickness flap was elevated through a para-crestal and two vertical incisions. A partial thickness flap was carefully reflected and apically displaced to achieve a recipient site free of muscle fiber attachment. With great care, the flap was sutured to fix its margins and base by a simple interrupted periosteal suture in the new apical position using a 6/0 non-resorbable suture (Proline, Ethicon, USA).

 The lingual side of the crestal incision was prepared by de-epithelization of the crestal keratinized mucosa to allow healing with either an onlay free gingival graft or a PRF membrane. Tissues covering the implant were excised to remove the implant cover screw.

**Figure 2:**
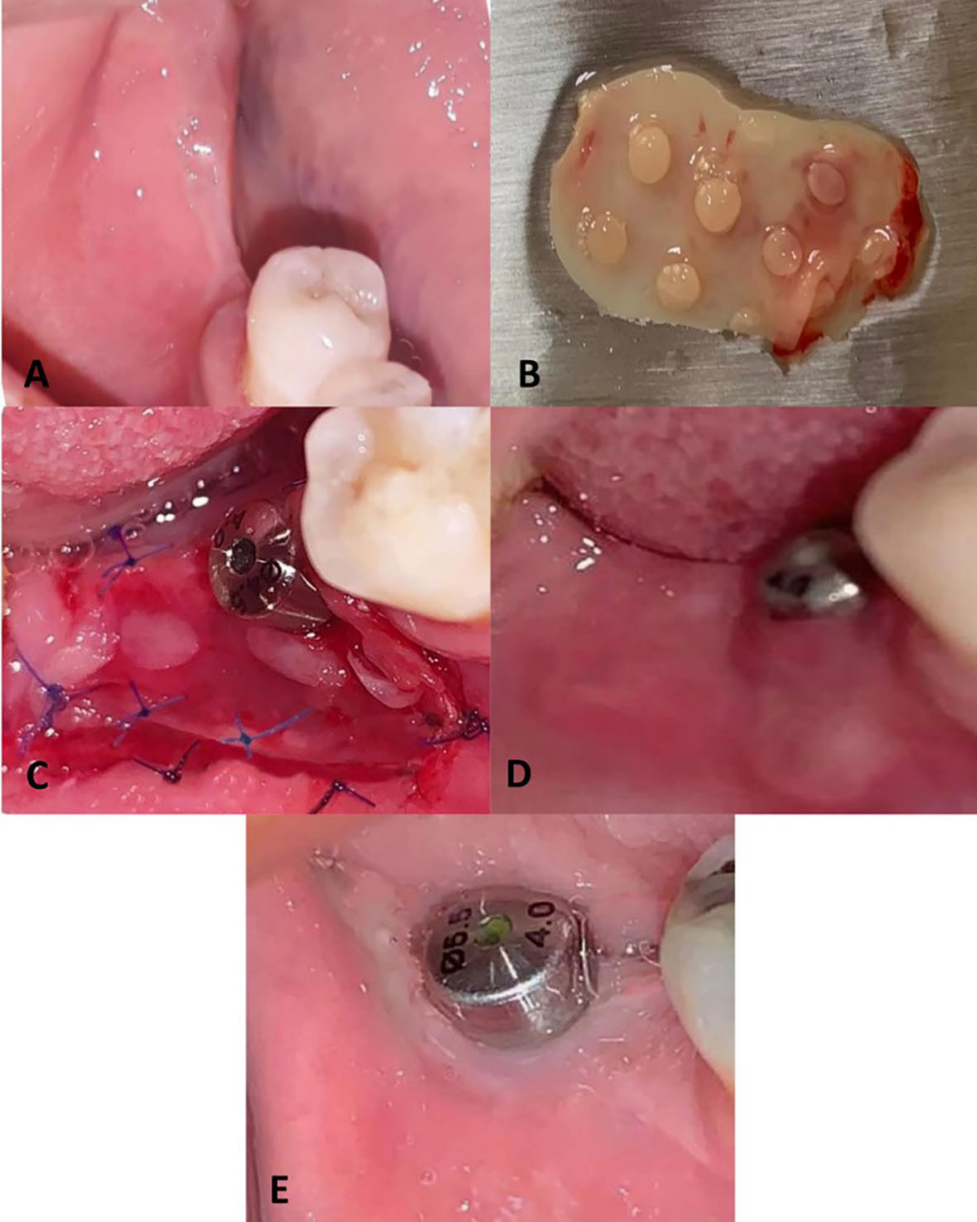
PRF onlay graft A: pre-operative FFG side B-free onlay PRF graft C- PRF onlay graft fixed with healing abutment and periosteal sutures D-healing and formation of keratinized mucosa after 1 month E- keratinized mucosa after 3 months postoperatively.

 On the study side, an onlay PRF membrane was prepared following the protocol described by Choukroun et al.^8^ Ten milliliters of whole venous blood were collected into two sterile 10-mL PRF preparation tubes. The tubes were inserted into the centrifuge machine running at 3,000 rpm for 10 minutes. The blood was separated into three layers; an upper straw-colored acellular plasma, a middle fraction containing the fibrin clot, and a red-colored fraction containing red blood cells at the bottom of the tube. The PRF was grasped with a tissue forceps and separated from the inferior layer by scissors. PRF membrane was obtained by using a “PRF box” to control membrane thickness. PRF membrane was carefully delivered to the surgical site over the whole recipient bed and extended slightly lingually and more buccally. The membrane was then fixed by interrupted periosteal sutures all around using a 6/0 non-resorbable proline suture. After membrane fixation, a small punch of the membrane was carefully placed over the implant and gingival healing abutment to avoid membrane wrinkling (Figure 2).

 On the control side, a graft of approximately 15‒20 mm in length and 5‒15 mm in width was harvested from the palate. A band of 2‒3 mm was left around the gingival margin of the teeth to avoid recession; then, the graft was harvested from the first molar–canine area. Two vertical incisions (anterior and posterior) and a horizontal incision (occlusal) were made perpendicular to the surface, with a depth of 1.5‒2 mm using a #11 scalpel blade. The edge of the graft was slowly elevated using tissue forceps and gently separated from the palate. The graft containing the epithelium and a thin layer of connective tissue was obtained from the donor site with a thickness of approximately 1.5‒2 mm. The harvested graft was soaked in isotonic saline solution and kept on wet sterile gauze. Hemostasis (blood clot) was achieved by applying pressure with sterile gauze. The palatal donor site was covered with a surgical gel foam pack.

**Figure 3:**
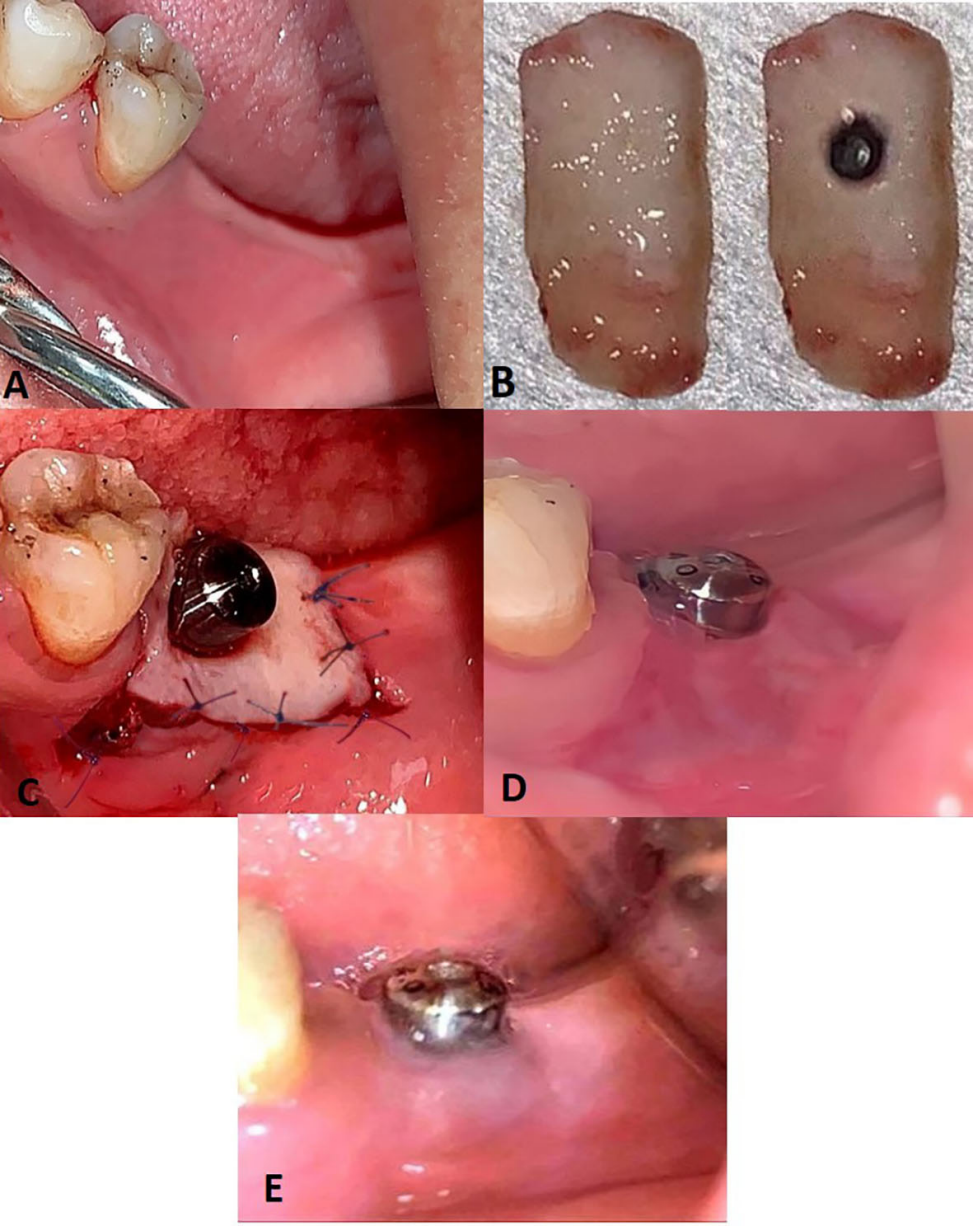
Free gingival onlay graft A: pre-operative FFG side B-free onlay graft harvested from the palate C-free gingival onlay graft fixed with healing abutment and periosteal sutures D- healing and formation of keratinized mucosa after 1 month E- keratinized mucosa after 3 months.

 A perforation was made by a rubber dam puncture at the position of the healing abutment for firm fixation around the implant. The onlay FGG was fixed using healing abutment and interrupted periosteal sutures all around by a 6/0 non-resorbable proline suture (Figure 3).

 All the subjects were prescribed systemic antibiotics, analgesics, and 0.12% CHX mouthwash for seven days. In addition, the subjects were instructed to maintain oral hygiene and have a soft diet for 24 h. They were recalled after 7‒10 days for removing the sutures.

 Three months later, after complete gingival healing, an indirect implant-level impression was taken for the implants using the closed tray impression technique to fabricate the final crowns. After crown fabrication was completed, the healing abutment was unscrewed, and the prosthetic abutment was placed and screwed using a toque wrench with 25-N force. Then the crown was checked and cemented using a dual-cured resin cement.

###  Clinical evaluation

 To attain intra-examiner reproducibility, six subjects were assessed twice in one visit over a one-hour interval for the clinical periodontal indices used in this study. The second assessment was carried out blindly relative to the first one. The reproducibility of the data was determined by calculating the number of the sites examined, where the scores were repeated exactly or to an accuracy of ±1 mm for each site (weighted kappa values: intra-examiner reproducibility was 0.87).

###  Assessment of the width of keratinized mucosa

 Clinical evaluation of keratinized gingiva was performed by measuring the distance from the gingival crest to the mucogingival junction using a calibrated periodontal probe. Measurements were made one month and three months after the second-stage surgery.

###  Evaluation of shrinkage of grafted tissue

 Vertical and horizontal dimensions of the grafted tissue were measured, and the surface areas were calculated (by multiplying the width by the length) for both study groups immediately after augmentation procedures. Measurements were repeated one month and three months later. All measurements were compared, and shrinkage percentages were calculated for each study group.

###  Evaluation of peri-implant soft tissue health

 Peri-implant soft tissue health was evaluated using the modified plaque index (MPI),^11^modified gingival index (MGI),^12^ and peri-implant probing depth (PD)^13^ one month and three months after the second-stage surgery on both sides of the groups.

###  Radiographic evaluation

 Digital periapical x-rays were made by the paralleling technique immediately after implant placement and three months after the second-stage surgery to assess the bone level around the implant. Vertical marginal bone level measurement was made from the implant–abutment interface to the first bone‒implant contact.^14^ The x-rays were analyzed by the ClearDent program to measure the distance between bone level and implant collar.

###  Statistics

 Data entry and statistical analyses were performed using SPSS 16.0 (SPSS Inc., Chicago, IL, USA). Data were first tested by the Shapiro-Wilk test for the distribution of data. Parametric data were expressed in means and standard deviations. Independent t-test was used to compare two means in different groups.

## Results

 Fifteen patients (8 females and 7 males) were included in the study. Their age range was from 25–35 years. Thirty implants were placed. The distribution of the replaced missing teeth bilaterally in each patient were 8 mandibular second premolars, 18 mandibular first molars, and 4 mandibular second molars.

 The width of keratinized mucosa in both groups was assessed 1 and 3 months after the augmentation procedures. There was a significant increase in KMW after 1 and 3 months in both groups (P<0.05). In addition, the width of keratinized mucosa in the FGG group was significantly higher than that in the PRF group after 1 and 3 months (P=0.0035 and P=0.0035, respectively). Moreover, there was no significant increase in the keratinized mucosa width in each group when KM width was compared after 1 and 3 months (P>0.05) (Table 1).

**Table 1:** Comparison of keratinized mucosal width between the study groups

**Keratinized mucosa width (mm) both groups (P-value) **	**Study group (PRF) **	**Control group (FGG) **	**Test of significance between **
**Baseline (mean ± SD) **	1.83±0.23	1.8±0.22	P=0.163
**After 1 month (mean ± SD) **	5.86±0.37 ^a^	8.67±0.74 ^a^	P=0.0028*
**After 3 months (mean ± SD) **	6.58±0.45 ^a^	9.16±1.64 ^a^	P=0.0035*

t: Student’s t-test *statistically significant between both groups ^a^ is statistically significant as compared to baseline. Statistically significant difference if P<0.05. PRF: platelet-rich fibrin FGG: free gingival graft

 A comparison of the shrinkage percentage of both PRF and FGG 1 and 3 months after augmentation showed a significantly higher mean shrinkage percentage in the PRF group compared to the FGG group (P<0.001). However, at different study intervals in the same group, there was no significant difference between 1- and 3-month intervals (P>0.05) (Table 2).

**Table 2:** Comparison of shrinkage percentages of the graft between the study groups

**Shrinkage percentage ** **both groups (P-value) **	**Study group (PRF) **	**Control group (FGG) **	**Test of significance between **
**After 1 month (mean ± SD) **	43.53±7.73	14.89±4.57	P<0.001*
**After 3 months (mean ± SD) **	45.96±5.94	15.01±3.87	P<0.001*

t: Student’s t-test, *statistically significant between both groups Statistically significant difference if P<0.05. PRF: Platelet rich fibrin FGG: Free gingival graft

 A comparison of the PRF and FGG groups regarding peri-implant soft tissue health 1 and 3 months after augmentation revealed no significant differences between the two groups in plaque and gingival indices or periodontal probing depths. There was no significant difference in the same study group at different study intervals. In group 1, no significant difference was found in PI, GI, and PD at different time intervals (P=0.44, P=0.08, and P=0.09, respectively). In group 2, no significant differences were found in PI, GI, and PD 1 and 3 months after graft augmentation (P=0.24, P=0.16, and P=1.97, respectively). Regarding the peri-implant bone level, there was no significant difference between the two groups regarding mesial and distal bone levels (Table 3).

**Table 3:** Comparison of periodontal indices and bone loss between the study groups

	**Study group (PRF) **	**Control group (FGG) **	**Test of significance between ** **both groups (P value) **
PI (mean ± SD) 1 month 3 months	0.58±0.75 0.55±0.32	0.55±0.66 0.42±0.33	P=0.437 P=0.35
GI (mean ± SD) 1 month 3 months	0.59±0.79 0.31±0.27	0.55±0.5 0.38±0.32	P=0.425 P=0.32
PD/mm (mean ± SD) 1 month 3 months	2.66±0.37 2.33±0.37	2.78±0.38 2.5±0.41	P=0.382 P=0.258
Bone Loss (mean ± SD) Mesial BL Distal BL	0.183±0.075 0.167±0.05	0.183±0.07 0.150±0.055	P=1.0 P=0.599

t: Student’s t-test Statistically significant difference if P<0.05 PRF: Platelet-rich fibrin FGG: free gingival graft PI: plaque index GI: gingival index PD: probing depth

## Discussion

 Traditionally, sufficient keratinized gingiva has been recognized to maintain healthy gingival tissues and prevent gingival recession. Notably, it is believed that the success of implants depends on the ability of the mucosa to play its biological protective role between the oral environment and the implants.^15^

 The appropriate timing for gingival augmentation surgery around a dental implant is a matter of controversy. This study evaluated the gingival augmentation procedures during second-stage surgery, as many reports show it can be performed mostly during stage 2 surgery or postprosthetic treatment periods.16,17^16,17^

 Soft tissue augmentation at the time of implant insertion may lead to tissue laceration, especially in thin gingival biotype, and it may cause more discomfort to the patient because of the lengthy surgery.^18^ Moreover, Stimmelmayr et al^19^ reported that the amount of shrinkage of the FGG was greater in the group receiving augmentation simultaneously with implant placement; however, the difference was not statistically significant.

 Another research showed that other criteria for augmentation surgery timing, such as VBH (vertical bone height), the number of implants, and muscle hyperactivity (high muscle attachment, HMA), were highly important. When the number of implants is >2, and there is HMA, surgical procedures should be performed before or during the second surgical procedure to avoid any complications that might develop due to insufficient KMW (such as the rupture of flap due to stretching) or to prevent the development of peri-implant mucositis/peri-implantitis.^20^

 In this study, an onlay graft technique was used because it has several advantages. It is not used only to treat mucogingival problems like inadequate width and thickness of attached gingiva and implant coverage procedures during the same surgical intervention; it can also increase ridge height in case of deficient ridges, as reported by Prathap et al^21^ (2013). Moreover, this technique is simple and does not require expensive biomaterials as the graft can be taken from the patient’s oral cavity. The onlay technique might increase the tight connection of tissues surrounding the implant, improving tissue resistance from the augmented KM over time.^22^

 This study evaluated the increase in KM width following different augmentation grafts; the KM width increased significantly using FGG and PRF. FGG sides showed a significantly higher KM width than the PRF sides. The free gingival graft (FGG) is still the gold standard of care primarily because of its high level of success.^23,24^

 Moreover, this study showed that PRF significantly increased the KM width after 1 and 3 months compared to the baseline, consistent with a study by John Wiley et al^25^ (2018), who showed that the PRF membrane might increase the width of the keratinized mucosa around dental implants.PRF created a sufficient amount of keratinized tissue with a minimally invasive technique compared to the KMW at baseline and 3 months after augmentation. It has low cost and low patient morbidity.^26^ Another research had the same opinions about PRF’s ability for soft tissue regeneration.^27^ However, the results of this study were different from those reported by Naik et al^28^ and Hehn et al,^29^ who assessed the PRF membrane at a 3-month follow-up and found that vertical dimensions for KM data were not statistically significant.

 The significant increase in the width of the attached gingiva around the implant using the PRF membrane can be explained by the preparation of PRF. It is a simple and inexpensive procedure to form an autologous fibrin matrix containing many growth factors such as PDGF, TGF- β, VEGF, and epidermal growth factor. In addition, it serves as a scaffold for the migration and proliferation of epithelial cells.^9^ The natural fibrin framework protects growth factors from proteolysis; therefore, they can stay active for a longer period (up to 28 days).^30^ This leads to effective neovascularization and accelerated wound closure with a lower incidence of postoperative infections.^8,9^ The adhesive, mechanical properties, and the fibrin glue function of the PRF membrane play an important role in avoiding infection and inflammation at the donor site of the operating field.^31^

 In the present study, the shrinkage was significantly different between FGG and PRF at 1- and 3-month follow-up intervals, which is different from the results of other studies with 6-week follow-up periods.^21,32^ In addition, in this study, most of the shrinkage of both FFG and PRF grafts occurred during the first month, consistent with another research showing that the majority of shrinkage occurred in the first month after surgery.^24^ With FGG, shrinkage primarily occurred during the first 28 days. However, after 30 days, no further shrinkage could be seen.^33^

 In this study, concerning the assessed periodontal indices around implants, the PRF group showed no significant difference compared to those in the FGG sides at 1- and 3-month follow-ups, which is different from a study by Schmitt et al.^24^ The insignificant difference in clinical periodontal indices between both groups might be explained by the significant increase in the width of KM around implants obtained by both FFG and PRF compared with the baseline. Good oral hygiene can be achieved easily around dental restorations surrounded by an adequate band of keratinized gingival tissue.^23^ Therefore, to achieve stable peri-implant health, it is important to achieve a proper soft tissue seal around dental implant/restorations.^4,15^

 Several studies have investigated the effect of soft tissue augmentation around dental implants and marginal bone loss. Several animal studies have reported that a combination of plaque accumulation and injury to the “biologic width” could result in crestal bone loss around implants.^34,35^ However, in another human study, available evidence only focused on improvements in clinical parameters rather than investigating changes in crestal bone level before and after augmentation of KM by FGG.^32^

 According to the results of this study, the baseline for radiographic bone level measurements was immediately after implant placement (T0). These measurements were compared with the bone level measurements 3 months after the second-stage surgery (T1). A certain amount of peri-implant bone resorption was found in the two groups.

 When changes in the crestal bone level at implant placement and six months later were compared with the baseline at the mesial and distal sides of the implants, bone loss was detected in both groups, with no significant difference between the two groups. Therefore, there was no significant effect on bone level surrounding the dental implant after increasing the KM width by FGG or PRF membrane.

 This finding was confirmed by other studies ^29,36,37^ that reported almost no effect of FGG or PRF on peri-implant marginal bone levels.Other studies have an opposite opinion favoring soft tissue augmentation by FGG, indicating a benefit as they found that the crestal bone loss around dental implants significantly decreased in the FGG group in the 6-month follow-up after the implant was loaded.^38-40^

## Conclusions

 Within the limitations of this study, it can be concluded that PRF can increase the keratinized mucosa width around dental implants. It has some advantages, including decreasing surgery time, ease of manipulation, and decreasing postoperative pain. However, FGG has a significantly higher ability to augment keratinized mucosa around dental implants.

## Acknowledgments

 None.

## Competing Interests

 The authors declare that they have no competing interests related to authorship and/or publication of this work.

## Authors’ Contributions

 ZMD: Editing, data collection, statistics, SM: Reviewing, periodontal surgery, editing, ASS; Reviewing, implant surgical procedure, editing, BM: Editing, reviewing, statistics.

## Funding

 The authors received no financial support for the research, authorship, and/or publication of this article.

## Availability of data

 The datasets used and/or analyzed during the current study are available from the corresponding author on reasonable request.

## Ethics Approval

 The protocols used in this research were approved by the Ethics Committee of Faculty of Dentistry, Mansoura University [IRB Protocol No: 14060318].
